# Global raster dataset on historical coastline positions and shelf sea extents since the Last Glacial Maximum

**DOI:** 10.1111/geb.13573

**Published:** 2022-08-14

**Authors:** Johannes De Groeve, Buntarou Kusumoto, Erik Koene, W. Daniel Kissling, Arie C. Seijmonsbergen, Bert W. Hoeksema, Moriaki Yasuhara, Sietze J. Norder, Sri Yudawati Cahyarini, Alexandra van der Geer, Hanneke J. M. Meijer, Yasuhiro Kubota, Kenneth F. Rijsdijk

**Affiliations:** ^1^ Institute for Biodiversity and Ecosystem Dynamics University of Amsterdam Amsterdam The Netherlands; ^2^ Biodiversity and Molecular Biology Edmund Mach Foundation Research and Innovation Centre San Michele All'Adige Italy; ^3^ Faculty of Agriculture Kyushu University Fukuoka Japan; ^4^ Group Atmospheric Modelling and Remote Sensing Swiss Federal Laboratories for Materials Science and Technology Saint Gallen Sankt Gallen Switzerland; ^5^ Taxonomy and Systematics Group Naturalis Biodiversity Center Leiden The Netherlands; ^6^ Groningen Institute for Evolutionary Life Sciences University of Groningen Groningen The Netherlands; ^7^ Area of Ecology and Biodiversity, Swire Institute of Marine Science, Institute for Climate and Carbon Neutrality, Musketeers Foundation Institute of Data Science, and State Key Laboratory of Marine Pollution School of Biological Sciences, University of Hong Kong Hong Kong SAR China; ^8^ Environmental Science Group Copernicus Institute of Sustainable Development, Utrecht University Utrecht The Netherlands; ^9^ Research Centre for Climate and Atmosphere National Research and Innovation Agency Republic of Indonesia (BRIN) Bandung Indonesia; ^10^ Vertebrate Evolution, Development and Ecology Naturalis Biodiversity Center Leiden The Netherlands; ^11^ Department of Natural History University Museum of Bergen Bergen Norway; ^12^ Human Origins Program, National Museum of Natural History Smithsonian Institution Washington District of Columbia USA; ^13^ Biology Program Nishihara, Faculty of Science University of the Ryukyus Nishihara Japan

**Keywords:** coastline retreat, connectivity change, glacial sensitive model, insular biodiversity patterns, palaeogeography, Pleistocene climate change, prehistorical human settlement, sea‐level fluctuations, shelf expansion

## Abstract

**Motivation:**

Historical changes in sea level caused shifting coastlines that affected the distribution and evolution of marine and terrestrial biota. At the onset of the Last Glacial Maximum (LGM) 26 ka, sea levels were >130 m lower than at present, resulting in seaward‐shifted coastlines and shallow shelf seas, with emerging land bridges leading to the isolation of marine biota and the connection of land‐bridge islands to the continents. At the end of the last ice age, sea levels started to rise at unprecedented rates, leading to coastal retreat, drowning of land bridges and contraction of island areas. Although a growing number of studies take historical coastline dynamics into consideration, they are mostly based on past global sea‐level stands and present‐day water depths and neglect the influence of global geophysical changes on historical coastline positions. Here, we present a novel geophysically corrected global historical coastline position raster for the period from 26 ka to the present. This coastline raster allows, for the first time, calculation of global and regional coastline retreat rates and land loss rates. Additionally, we produced, per time step, 53 shelf sea rasters to present shelf sea positions and to calculate the shelf sea expansion rates. These metrics are essential to assess the role of isolation and connectivity in shaping marine and insular biodiversity patterns and evolutionary signatures within species and species assemblages.

**Main types of variables contained:**

The coastline age raster contains cells with ages in thousands of years before present (bp), representing the time since the coastline was positioned in the raster cells, for the period between 26 ka and the present. A total of 53 shelf sea rasters (sea levels <140 m) are presented, showing the extent of land (1), shelf sea (0) and deep sea (NULL) per time step of 0.5 kyr from 26 ka to the present.

**Spatial location and grain:**

The coastline age raster and shelf sea rasters have a global representation. The spatial resolution is scaled to 120 arcsec (0.333° × 0.333°), implying cells of *c*. 3,704 m around the equator, 3,207 m around the tropics (±30°) and 1,853 m in the temperate zone (±60°).

**Time period and temporal resolution:**

The coastline age raster shows the age of coastline positions since the onset of the LGM 26 ka, with time steps of 0.5 kyr. The 53 shelf sea rasters show, for each time step of 0.5 kyr, the position of the shelf seas (seas shallower than 140 m) and the extent of land.

**Level of measurement:**

Both the coastline age raster and the 53 shelf sea rasters are provided as TIFF files with spatial reference system WGS84 (SRID 4326). The values of the coastline age raster per grid cell correspond to the most recent coastline position (in steps of 0.5 kyr). Values range from 0 (0 ka, i.e., present day) to 260 (26 ka) in bins of 5 (0.5 kyr). A value of “no data” is ascribed to pixels that have remained below sea level since 26 ka.

**Software format:**

All data processing was done using the R programming language.

## 1 INTRODUCTION

**FIGURE 1:**
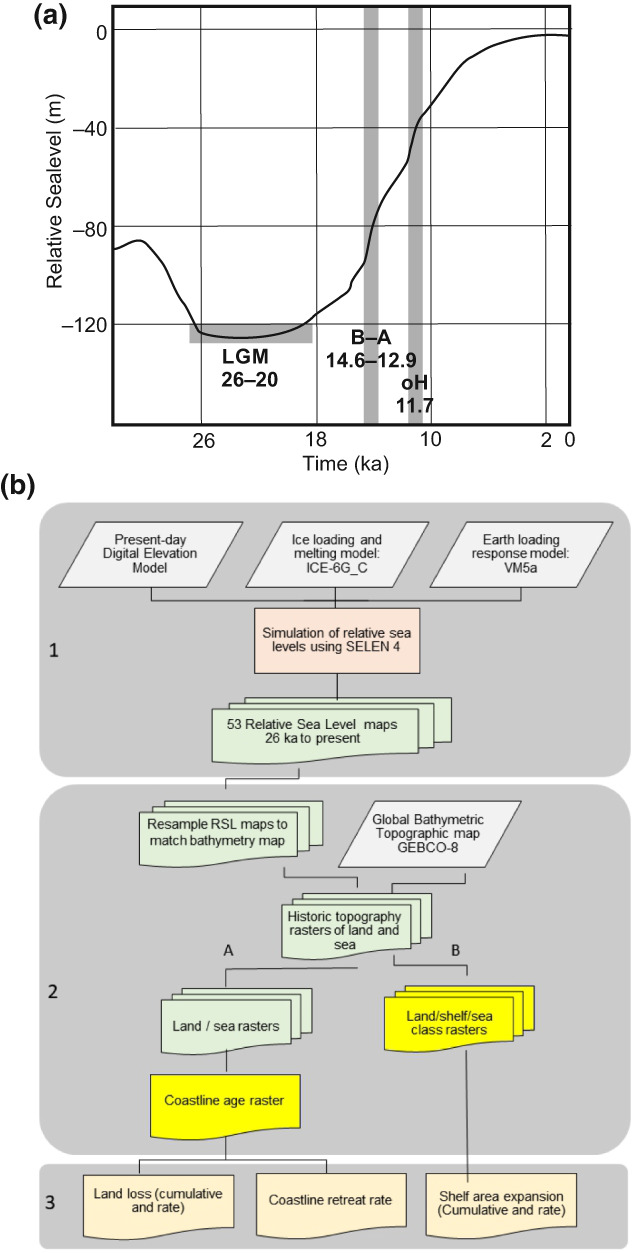
(a) Sea‐level rise curve over 26 kyr. LGM is the Last Glacial Maximum period. Sea‐level rise accelerated during the Bölling–Allerød (B‐A) interstadial period (14.6–12.9 kyr bp) and at the onset of the Holocene (oH) epoch (11.7 kyr bp). (b) Workflow diagram showing the steps to obtain the historical coastline position raster. In order to produce per time step of 0.5 kyr relative sea‐level positions on a global scale, we use the SELEN^4^ relative sea‐level model. In step 1, we use a global digital elevation model, the output of the ice‐loading model ICE_6GC and the output of a loading response model, VM5a, as input for the SELEN^4^ model. The SELEN^4^ model combines the inputs of step 1 iteratively to calculate relative sea levels per raster cell based on the global melt water distribution, the response of the Earth's crust to seawater loading effects, crustal bouncing attributable to glacial loading effects and the effects of the gravity field of the ice sheets pulling up seawater. The output of SELEN^4^ is 53 rasters, with raster cells showing the relative position of sea level per time step, in comparison to the present. In step 2, to produce historical topography rasters, each relative sea‐level position raster per time step is subtracted from the present‐day topography raster. Before doing this, to match the rasters they must be resampled to a finer resolution. The output is 53 historical topographic rasters. In step 2A, the 53 historical topography rasters are classified in land/sea Boolean rasters, after which the 53 Boolean rasters are aggregated into a coastline age map. In step 2B, the 53 historical topography maps are reclassified into 53 Boolean land/shelf/sea maps using three classes (“no data” are deeper than 140 m; “1” is shallower than 140 m; and “0” is island). In step 3, based on the historical coastline and land/shelf/sea maps, the cumulative values and rates for shelf expansion, land loss and coastal retreat are calculated.

Present‐day marine and terrestrial biodiversity and their evolution in the tropical and subtropical realms have been influenced by historical sea‐level change (Hewitt, 2000; Hoeksema, 2007; Huang et al., 2018, 2019; Norder et al., 2019; Weigelt et al., 2016; Woodruff, 2010). The amplitude of sea‐level fluctuations increased from *c*. 3 Ma and culminated in >130 m amplitude over the last 26 kyr (Lambeck et al., 2014; Figure 1a). Especially in the (sub)tropical zone, such sea‐level fluctuations have shaped the morphology of reefs and the position of river mouths, caused major disruptions in sea‐current regimes and formed and drowned land bridges, thereby affecting the dispersion and connectivity of both marine and terrestrial biota, including humans (Bover et al., 2008; Cacciapaglia et al., 2021; Chiu et al., 2017; Fernández‐Palacios et al., 2016; Hanebuth et al., 2000, 2011; Meijer et al., 2010; Oppenheimer, 2009; Van den Bergh et al., 2001; Voris, 2000; Yasuhara et al., 2017). The biogeography of marine biota and their evolution is therefore highly influenced by the spatio‐temporal dynamics of coastlines, shelf areas and connectivity of seaways (Hanebuth et al., 2011). During glacials, extensive areas of shelf floors were exposed, causing a reduction in marine shelf habitat (Avila et al., 2019). Consequently, marine biota locked in isolated pools generated by emerged land bridges experienced higher degrees of genetic isolation (Hoeksema, 2007). With rapidly rising sea levels, ≥100 m at the end of the glacial period, shallow marine habitats were displaced over major distances to reach their present‐day positions (Cacciapaglia et al., 2021; Hoeksema, 2007; Veron et al., 2009). This is likely to have contributed to local extinctions of species that were unable to keep up with sea‐level rise and resulted in both relict and refuge communities (Hoeksema, 2007; Veron et al., 2009).

A key period to study the effects of coastline dynamics is the last 26 kyr, which begins with the onset of the Last Glacial Maximum (LGM) period, when ice sheets reached their largest extents and when sea levels dropped to their lowest position, at 135 m below present. Owing to rapid global warming at the end of the last ice age, sea levels rose and achieved rates >40 m/kyr, the highest sea‐level rise rates over the last 5 Myr (Lambeck et al., 2014). Consequently, shelf area expansion rates and coastline retreat rates were among the highest. The rapid spatio‐temporal changes of both the total area and the location of shelf sea areas and coastlines must have had profound effects on the distribution of biotas and their gene pools. The period of sea‐level rise between 26 ka and the present is therefore crucial for understanding the present‐day distributions of marine and terrestrial species and to study micro‐evolutionary effects in organisms (Hammoud et al., 2020; Hoeksema, 2007, 2012; Macaulay et al., 2005; Veron, 1995). However, currently existing palaeogeographical workflows often do not reconstruct coastline positions correctly and might therefore yield imprecise estimates of the rates of coastline retreats, shelf expansions and land losses.

Most palaeogeographical workflows used in biogeographical and archaeological studies reconstruct historical coastlines by relating present‐day seafloor depth directly to a past global sea‐level position (e.g., Athanassiou et al., 2019; Cacciapaglia et al., 2021; Norder et al., 2019; van der Geer et al., 2016, 2017; Voris, 2000; Weigelt et al., 2016; Woodruff, 2010; Yasuhara, 2008). These studies assume that the sea floor remains static under changing water loads and that gravitational effects of ice sheets on the sea levels are absent. In reality, sea levels are adjusting dynamically to geophysical changes that include the depression of the Earth's crust under seawater loading, the pulling‐up effect of sea water by the gravitational forces of the ice sheets, and the redistribution of sea water on the planet (Gehrels, 2010). For correct coastline reconstructions, glacio‐isostatic models should be used that take both the elasticity and the viscosity of the Earth's crust and mantle into account, in addition to the spatio‐temporal distribution of the palaeo‐ice sheets and their effects on the Earth's crust and sea water depths (Gehrels, 2010; Lambeck et al., 2014; Spada, 2017). Ignoring these factors leads to incorrect palaeo‐coastline positions and incorrect derived metrics, such as coastline retreat rates and shelf expansion rates.

Here, we present a novel global coastline age raster, based on a state‐of‐the‐art geophysical workflow (Figure 1b), which records the age of coastline positions since the LGM. The coastline raster allows calculations of both global and regional rates of coastal retreat over the last 26 kyr in time steps of 500 years, in addition to the expansion rates of shelf areas (with a depth <140 m). The raster can be used to reconstruct regional palaeo‐coastline positions, to calculate the rate of shelf area expansion and to quantify coastline retreat over the last 26 kyr. We describe primary data sources and present derived metrics including coastal retreat rates, landloss rates and shelf sea area expansion rates for the whole subtropical and tropical realm (30°N and 30°S) and for three selected case studies. We focus on the (sub)tropical realm because this area contains the main global hotspots of both marine and terrestrial biodiversity and was pivotal for human migrations. Moreover, this area is located far away from the continental ice sheets and thus lacks the added complexity of ice‐sheet interactions (Milne & Mitrovica, 2008). We discuss the reliability of both raster datasets and steps for future improvements.

## 2 METHODS

We developed a workflow (Figure 1b) that includes the six preprocessing and analysis steps necessary to produce the coastline age raster and the 53 rasters with shelf sea <140 m (land/shelf/sea raster) and to calculate three derived metrics: shelf area expansion rate, cumulative land loss and coastline retreat rate. In our analysis, we consider the shallow continental marine shelf zone as a marine zone shallower than 140 m, which coincides roughly with the better‐aerated photic zone.

In the first step, we used the open‐source SELEN^4^ program that simulates geophysical effects on global sea levels and calculates spatio‐temporal relative sea‐level (RSL) rasters (Spada & Melini, 2019). RSL rasters show the differences in sea depth and topography of a selected time stamp in comparison to the present. SELEN^4^ provides as output rasters that indicate how much the crust was depressed or uplifted by the loading of ice sheets and meltwater that is added to the oceans. In addition, the raster values also incorporate the effects of the gravitational forces exerted by the ice sheets and effects of the rotation of the Earth. SELEN^4^ comprises an ice model, ICE‐6G_C, and a viscoelastic Earth model, VM5a (Argus et al., 2014). We used as Earth surface model a global high‐resolution digital elevation model (DEM including both bathymetry and topography), DEMSRE3a (Hengl, 2018). The DEM was subsampled at a resolution of 100 Tegmark discretization pixels, which corresponds to roughly equilateral triangles with sides of 38 km, globally (Tegmark, 1996). The model was run up to spherical harmonic order 192. The use of Tegmark pixelation factor 100, spherical harmonic order 192, and ice model ICE‐6G would be referred to as a model run “R100/L192/I6” in the terminology of SELEN^4^ (Spada & Melini, 2019). Fifty‐three rasters were generated that present RSL positions per raster cell and per time step of 500 years over a total period of 26 kyr. The output rasters were created on a regular latitude/longitude raster with steps of 0.2° × 0.2°. Owing to the low variation of values between raster cells (which was <1.5 m), this was a sufficient resolution.

In the second step, we combined the 53 RSL rasters with a global integrated topographic and bathymetric raster (cell size 0.0333° × 0.0333°) provided by General Bathymetric Chart of Oceans (GEBCO Compilation Group, 2019, 2021). The RSL output was resampled to match the high‐resolution bathymetry raster using a bilinear interpolation. We then combined the RSL and bathymetry datasets into 53 historical topography rasters. These historical topography rasters show both the sea‐level depths and the terrestrial elevation per 0.5 kyr time step. To produce the coastline age raster (step 2A), we separated the historical topographical rasters into Boolean land/sea rasters. For each 0.5 kyr time unit, a raster cell is assigned a value of either one (above sea level) or zero (below sea level). Next, the most recent time period at which a raster cell was land was extracted, and these cells were combined into a single coastline age raster. To produce the shelf sea rasters (step 2B), showing the extent of seas shallower than 140 m, we used the historical topography rasters to produce 53 rasters that were classified into land areas (one), shelf areas (zero) and deeper water (>140 m), here assigned as “no data”.

After preparing the coastline age raster and the 53 shelf sea rasters, derived metrics were calculated in the third step. The shelf sea rasters were used to calculate the cumulative shelf sea area expansions (in square kilometres) metric and the shelf sea expansion rate (in square kilometres per 0.5 kyr) metric. The coastline age raster served as input to calculate both the cumulative (in square kilometres) and rates (in square kilometres per 0.5 kyr) per raster cell of coastline retreat and land loss. As a last metric, we calculated the maximum horizontal coastline retreat rate (in metres per year) per 1° raster cell by identifying the drowned areas at a given time and their adjacent areas, which drowned during the next time step, and calculated their average distance.

The Figshare repository stores the coastline age raster and shelf sea rasters both derived from GEBCO 2019 (GEBCO Compilation Group, 2019) and GEBCO 2021 (GEBCO Compilation Group, 2021). Scripts are also made available to allow continuous integration of updated versions of the datasets with more recent GEBCO releases.

## 3 RESULTS

The novel coastline age raster shows the timing when parts of the global coastline were drowned after sea levels rose 26 ka (Figure 1a; Supporting Information Figure S1.1). The raster cells represent maximum ages (in thousands of years before present) of coastline positions. Plotting our geophysically reconstructed coastline ages of raster cells versus their present‐day water depths demonstrates that offsets of >60% exist, illustrating that equating depth with coastline age would lead to both erroneous palaeo‐coastline position assessments and erroneous quantification of coastal retreat rates (Supporting Information Figure S1.1). Deeper marine regions were flooded first, whereas shallower marine areas were flooded later.

**FIGURE 2:**
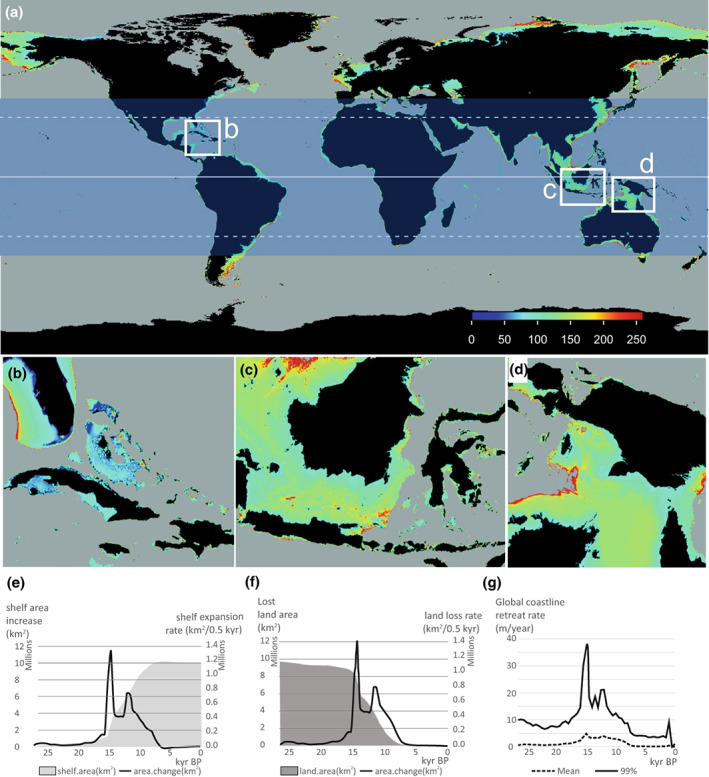
(a) Global map of historical coastline positions since 26 ka. The blue shaded zone is the (sub)tropical realm, between 30°N and 30°S, for which rates of increase in shelf sea area, land loss and coastline retreats are calculated (see below). Age of flooding is in thousands of years. Red is old (>20 kyr), blue is young (<10 kyr). (b) Caribbean region, illustrating the late drowning post‐10 kyr bp of shelfs around Cuba, the Bahamas and Florida. (c) East Sunda region, with most drowning between 20 and 15 kyr bp. (d) Australia and New Guinea, showing drowning of the shelf Gulf of Carpentaria ~18 kyr bp, while a land bridge extending from Australia Cape York peninsula (Australia) to New Guinea existed until *c*. 10 kyr bp. (e–g) Changes in the area of lands and marine shelfs (≥ −140 m) since 26 ka in the subtropical and tropical zone between ±30° latitude, with horizontal axes in thousands of years before present (blue zone in panel a). (e) Cumulative shelf sea area (in square kilometres) and rate of shelf sea expansion (in square kilometres per 0.5 kyr) at each time interval (black line; right axis). (f) Cumulative land loss curve (in square kilometres) and rate of land loss (in square kilometres per 0.5 kyr; black line; right axis). (g) Rate of horizontal coastline retreat (in metres per year) for each 1° raster cell in the (sub)tropical zone between −30 and 30°. Light grey and dark grey shaded curves represent average and 99th percentile per 500 year interval, respectively.

To illustrate this, we present a close‐up of three regions (Figure 2b–d): the Caribbean shelfs, Sunda shelfs and the Gulf of Carpentaria shelfs. The Caribbean shelf (Figure 2b) shows that major coastline retreats occurred only after 15 kyr bp, whereas the Sunda shelfs (Figure 2c) and Gulf of Carpentaria shelfs (Figure 2d) coastal retreats start shortly after the LGM, 20 ka. The maps also show that land bridges existed between most islands of the Bahamas, between Borneo and Sumatra and between Australia and New Guinea as recent as 10 ka. We calculated the extents of shelf sea expansion (≤140 m deep) and coastline retreat between the tropical and subtropical regions, −30°S and 30°N latitude. The shelf sea areas increased 10 times, from 10^6^ km^2^ during the LGM to 10^7^ km^2^ in the present day (Figure 2e), confirming that during the interglacial epochs, shallow marine habitats had reached their maximum extents (Avila et al., 2019). The highest shelf sea expansion rate of >1,3 × 10^6^ km^2^/0.5 kyr was realized at *c*. 14.0 ka, which comprised 12% of the present‐day shelf sea extent (Figure 2e). This rapid expansion was the result of a major melting pulse during the warming episode at the end of the last ice age, between 14.6 and 12.9 kyr bp (Figure 1a; Brendryen et al., 2020).

Shelf sea expansion went hand in hand with loss of terrestrial lands, including all tropical and subtropical lowland losses over the last 26 kyr, amounting to >9.7 × 10^6^ km^2^ (Figure 2f). Most of this loss occurred between 15 and 8 ka and involved drowning of lowland around islands and emerging shelfs. Between 7.5 and 16.5 ka, the land loss rate was >10^5^ km^2^/0.5 kyr, and loss rates peaked twice: between 14 and 14.5 ka, reaching >1.4 × 10^6^ km^2^/0.5 kyr, and again between 11.5 and 11.0 ka at *c*. 0.8 × 10^6^ km^2^/0.5 kyr. Within the tropical–subtropical region, the mean coastal retreat rates were 2–4 m/year between 15 and 8 ka, peaking at *c*. 4.8 m/year at *c*. 14.5 ka (Figure 2g). The expansion of shelf sea areas by 10^6^ km^2^ and drowning of land bridges led to the connectivity and expansion of marine biota and resulted in massive migration pulses and genetic exchange (Avila et al., 2019; Hoeksema, 2007; Veron et al., 2009). The commonly high coastal retreat rates of >10 m/year between 9 and 15 ka must have induced forced migrations of terrestrial biota, including humans, to higher grounds (Kealy et al., 2016; Oppenheimer, 2009) and, plausibly, formed the foundation of flood mythologies (e.g., Nunn, 2014; Ryan & Pitman, 2000). To illustrate the potential applications of the coastline age raster, we reconstructed the regional shelf sea and coastline dynamics driven by sea‐level rise since 26 kyr bp for archipelagos in the Caribbean Sea, the Sunda shelf and Banda Arc shelfs (Supporting Information Figure S1.2).

## 4 DISCUSSION

### 4.1 Applications

Sea‐level rise since the LGM 26 ka led to a massive expansion of shelf sea areas, coastal retreats and land losses globally, which resulted in major redistributions of marine and terrestrial biota. To study the consequences of such palaeogeographical changes for biodiversity, it is essential to use realistic reconstructions of shelf sea extents and best approximations of the rates of shelf sea expansion, coastal retreat and land loss.

Our new dataset is the first global raster with geophysically corrected coastline positions and shelf sea extents since the LGM. These data allow the calculation of global and regional shelf sea expansion rates, coastal retreat rates and land loss rates. The derived metrics we present are essential to assess the effect of a major sea‐level rise pulse on marine and terrestrial biota, their distribution and their genetic make‐up (Cacciapaglia et al., 2021; Hanebuth et al., 2011; Tian et al., 2020). The rasters also allow for identification of the precise timing of land‐bridge flooding, hence establishment of the timing of connectivity between marine gene pools or the timing of separation between terrestrial gene pools. The derived metrics can be used to assess the effects of historical spatial changes in coastline and shelf sea configuration on the distribution of biota and to assess whether present‐day species communities are the result of equilibria between migration and extinction rates (Avila et al., 2019; Fernández‐Palacios et al., 2016). Moreover, they can be used to test the species pump theory, which predicts that higher rates of connectivity and isolation lead to higher evolutionary rates (Gillespie & Roderick, 2014; Hoeksema, 2007; Qian & Rickleffs, 2012). They are also indispensable for testing relationships between species richness and island surface area (ISAR) and isolation (ISIR), especially in the context of extinction debt. Tan et al. (2022) developed an R package to assess the effects of palaeo‐coastline change of archipelagos on gene flow and patterns of community assemblages. Our model can be adapted to study the spatio‐temporal dynamics of marine habitats of specific depth ranges; for instance, coral seas habitats shallower than 40 m. The patterns emerging from the sea‐level rise since 26 kyr bp indicate which areas (shelf seas and islands) respond most radically to sea‐level rise. These locations are therefore most strongly influenced by sea‐level change to the highest degree, hence their biota might show high speciation and/or extinction rates. Finally, the metrics can be used to assess how rates and durations of palaeogeographical change, in terms of isolation, surface area and connection, affect micro‐evolutionary processes and rates.

### 4.2 Input datasets and accuracy

Although we used the most advanced sea‐level curve and global bathymetry to date, these models themselves might contain inaccuracies. A local validation study found that the base model of GEBCO 2021 (i.e., SRTM15_+ v.2.0) showed a general large topographical similarity to the observed bathymetry, but that the former was systematically underestimating the depth by 2.5 m and that artefacts appeared in regions shallower than 10 m below sea level (Foonde, 2019). This would imply that shelf sea reconstructions and timings of coastal drowning are overestimated (i.e., timing of drowning is more recent with GEBCO); the age inaccuracy depends on the rate of flooding, and the generated error will be larger for younger estimates of sea‐level rise effects after 7 ka, when rates of sea‐level rise had reduced to <1 m/kyr.

The GEBCO grid is integrating a range of bathymetric data sources with variable data quality, including highly accurate direct measurements (e.g., lidar, seismic and sounding) and less accurate indirect measurements (e.g., satellite‐derived gravity data and contour maps) (GEBCO Compilation Group, 2019, 2021). To obtain global coverage, interpolation and mathematical techniques are applied by GEBCO using the available source data. Consequently, the accuracy of the estimated depth might vary locally depending on the type of source bathymetry that is available within a region of interest (Tozer et al., 2019). For instance, although satellite altimetry allows missing data gaps to be filled, Tozer et al. (2019) found mean inaccuracies of 15 ± 180 m in depth when compared with sounding data in areas between the coastline and the continental rise. This could be expected to affect especially areas of the continental slope that are characterized by higher‐frequency and large‐amplitude depth variations, and to affect much less the shallow‐water regions near the coast, which are the regions of interest of the presented reconstructions (Tozer et al., 2019). Continuous efforts are made with GEBCO seabed 2030 project to collect highly accurate local bathymetric information, which will allow improvement of the GEBCO model and derived products (The Nippon Foundation‐GEBCO, 2022). To evaluate the source data and reliability of the bathymetry, GEBCO provides an additional grid, which includes the type identifier for each GEBCO grid cell (GEBCO TID; GEBCO Compilation Group, 2019, 2021). We therefore recommend using the GEBCO TID grid to evaluate whether GEBCO and the derived coastline age raster and shelf sea rasters are reliable for a specific region of interest.

With regard to the SELEN^4^ module, comprising a glaciation model and an Earth model, several model runs demonstrate that modelled local sea‐level curves (relative sea‐level curves) within the tropical realm reproduce local sea‐level curves reasonably well based on empirical data, with deviations generally being <10 m (Bagge et al., 2021: figure 5). Outside the tropical realms, the errors can be much higher owing to the complexity and various competing models used for the glaciation history in these areas. Accepting that relative sea‐level curves are reasonably well modelled, it can be expected that reconstructed coastlines, when not modified severely by erosion or sedimentation processes, are also realistic. One study in the Sicilian Channel, Italy, outside the subtropical realm, modelling coastline positions during the LGM (18–26 ka), found that the modelled coastlines could agree very well (<2 km) with empirically reconstructed coastlines based on seismic data interpretation (Lodolo et al., 2020: figure 6).

### 4.3 Improvements

Modelling did not include local effects on relative sea levels caused by regional tectonic change, regional geology (fracturing and faulting) and regional sedimentary processes (delta progradation, sedimentation and erosion). Geological vertical crustal motions (GVCM) can vary over time and space, with typical rates ranging between −1 and +1 mm/year. Over time spans of 10 kyr, uplift rates of 1 mm/year lead to vertical deviations of relative sea level >10 m. With the Sunda shelf subsiding at rates of 0.2–0.3 mm/year (Sarr et al., 2019), it subsided 5 m over the last 20 kyr, whereas the islands of Timor and Cuba, for example, were uplifted ~6 m over this time period (Peñalver et al., 2021; Vita‐Finzi & Hidayat, 1991). Correcting relative sea levels with GVCM rasters can have major repercussions for biogeographical assessments, especially when considering longer time spans (Husson et al., 2020; Kalb et al., n.d.). Our model can integrate locally composed GVCM raster files (in millimetres per year) to produce more realistic reconstructions. Delta progradations can also lead to different coastline configurations. When active, these geological processes can influence the reconstructed age of coastlines. Anomalies in the historical spatial coastline and shelf sea configuration can also occur owing to inaccuracies in the GEBCO gridded bathymetry dataset; for instance, as a result of missing sounding data in extensive shallower‐water (<2 m) regions south of the Bahamas, non‐existent islands appear (Figure 1b; Seabed 2030 Global Data Center, 2021). These inaccuracies can be mitigated by inclusion of updated bathymetry rasters including local, accurate bathymetric datasets.

In this paper, we analyse effects in the subtropical and tropical realms; data from the northern realms should consider local ice‐sheet configurations and melting dynamics. The time span of our historical coastline raster starts from 26 kyr bp to the present and is also constrained by the availability of the geophysical model SELEN^4^, which starts from 26 ka; when this model is updated to back model further in time, we can implement this in our modelling and study the dynamics of shelf expansion and coastal amplitudes over longer periods.

## BIOSKETCH

The study was led by the Biogeography & Macroecology (BIOMAC) laboratory in collaboration with the Computational Support (COMPSUP) team at the Institute for Biodiversity and Ecosystem Dynamics (IBED), University of Amsterdam. The central aim of BIOMAC is to quantify how biodiversity and abiotic components of the Earth system vary across space and time, how they interact, and how responses of species and ecosystems to changing environmental conditions can be predicted and forecast. Computational Support provides expertise in computational tools and data that are needed for scientific research at IBED.

## Supporting information


Appendix S1

## Data Availability

The data and scripts that support the findings of this study are available in Figshare at: https://doi.org/10.21942/uva.c.5754779.v1. The General Bathymetric Chart of the Oceans (GEBCO), SELEN^4^ and DEMSRE3a were derived from the public domain available at: GEBCO 2019: https://doi.org/10.5285/836f016a‐33be‐6ddc‐e053‐6c86abc0788e; GEBCO 2021: https://doi.org/10.5285/c6612cbe‐50b3‐0cff‐e053‐6c86abc09f8f; SELEN^4^: https://zenodo.org/record/3520451; and DEMSRE3a: https://zenodo.org/record/1637816.
