# Supplementary material for: Global raster dataset on historical coastline positions and shelf sea extents since the Last Glacial Maximum
Source: Glob Ecol Biogeogr. 2022 Aug 14;31(11):2162–71. doi: 10.1111/geb.13573 (PMC9804564; doi:10.1111/geb.13573)
Supplement: Supplementary file 1 — Appendix S1 [file GEB-31-2162-s001.pdf]

Appendix S1

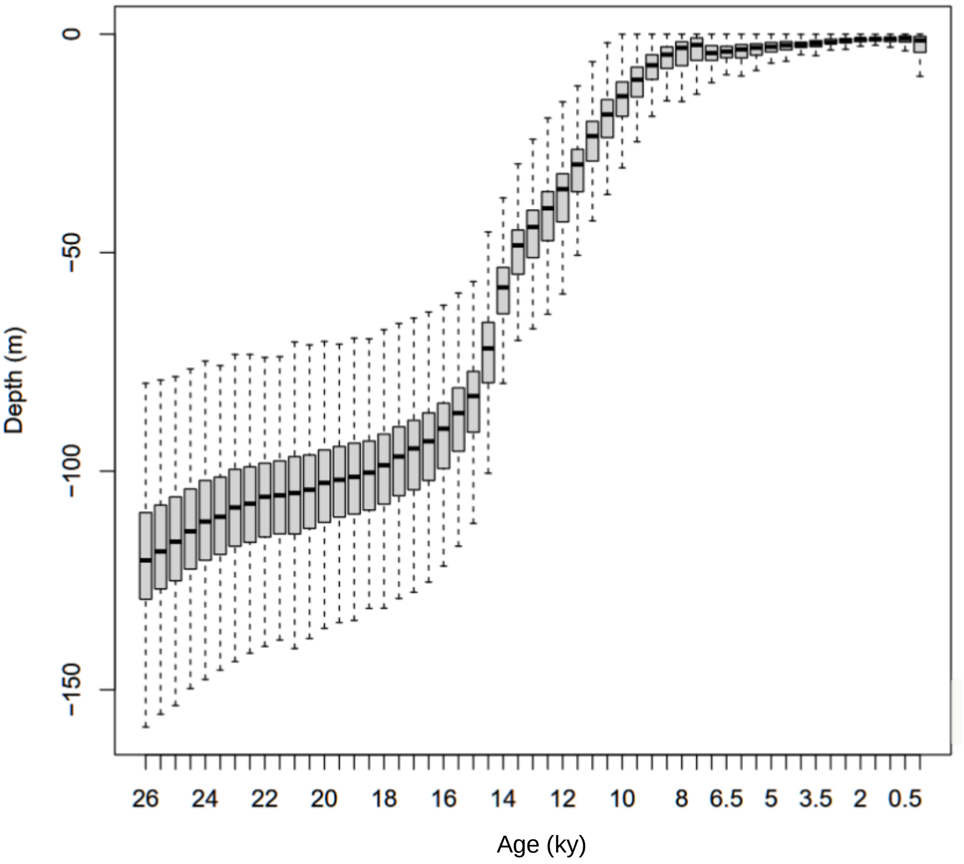

Figure S1.1. Age of coastline and water depth graph for the global subtropical region (30°S – 30N°). The boxes show the 25% to 50% quantile, and the whiskers show 1.5 * interquartile range in water depths per coastline age class. Note that outliers (i.e., larger or smaller than the 1.5 * interquartile ranges) are not shown. The graph shows that the assumption that the age of coastline varies in tandem with water depth is incorrect. Not using a geophysical workflow that includes relative sea level differences will lead to major erroneous reconstructions of coastline positions and rates of coastal retreats.

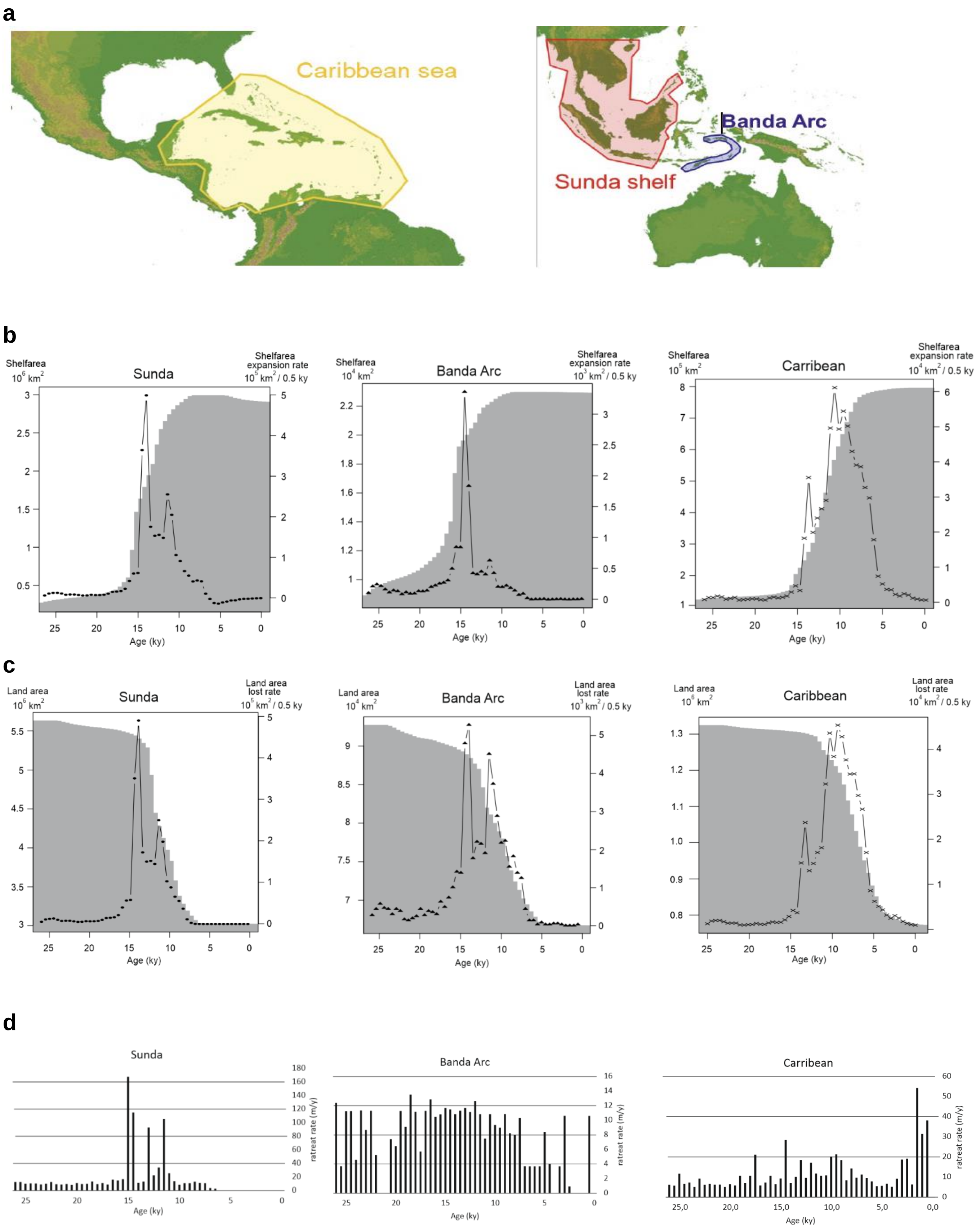
a
Caribbean sea
Banda Arc
Sunda shelf
b
Sunda
Banda Arc
Carribean
Shelfarea
Shelfarea expansion rate
Age (ky)
c
Sunda
Banda Arc
Caribbean
Land area
Land area lost rate
Age (ky)
d
Sunda
Banda Arc
Carribean
ratreat rate (m/y)
Age (ky)

Figure S1.2. To illustrate an application of the coastline age analysis we selected shelf regions in Sunda, Banda Arc shelfs and the Caribbean (Fig. 2b, c, d). These regions comprise major hotspots in both marine and terrestrial biodiversity.

Panel (a) Geographic setting and extent of analysis.

Panel (b) Shelf sea expansion curves with cumulative increase (bars: left axis) and rate of increase shelf areas in km^2 per 500 years (dots; right axis). Note that the reconstructions are not corrected for regional tectonic uplift or subsidence rates. Shelf seas expanded most radically in the Sunda region, with an over 11-fold growth from 2.6×10^5 km^2 to nearly 2.9×10^6 km^2. Achieving twice the size of the Gulf of Mexico, the Caribbean shelf seas expanded more than 6.5-fold from 1.2×10^5 to 8.0×10^5 km^2. The expansion of the Banda Arc shelf areas was over 2.5-fold, from 8.6×10^5 km^2 to 2.3×10^6 km^2. From the shelf sea expansion rate curves, it can be inferred that both Sunda and Banda Arc shelfs mostly expanded between 15 and 10 ky. The Sunda shelf sea expanded most radically with expansion rates spiking twice: firstly expanding ~500,000 km^2 / 500 y (which is the area equivalent of the present-day North Sea) at 14 ky, and secondly expanding ~250,000 km^2 / 500 y at 11.5 ky. To put things in perspective, the area expansion of the Sunda shelf sea at 15 ky amounted to twice the size of the Sunda shelf sea at the LGM. The second peak at 11.5 ky added an area equal to the size of the Sunda shelf sea at the LGM. At the Banda Arc, the shelfs expansion shows a single major peak of 3.3×10^5 km^2 / 500 y, which is about 40% of the original LGM size of the Banda Arc shelfs. Because of the shelf morphology the second later pulse around 11 ky in sea level rise did not so much affect the Banda Arc shelfs. The Caribbean shows a more extended period of shelf sea expansion between 15 and 5 ky. Two peaks are present: the highest of 6.0×10^4 km^2 at 11 ky, represents half of its minimum LGM size. A smaller peak of 3.5×10^4 km^2 / 500 y, again at 14 ky ago, represents about 30% of its LGM size. The more extended response in shelf sea expansion in the Caribbean can be explained by both the presence of extensive low-angle submarine platforms, and the different geophysical response in this shallow shelf, closer to the Northern American ice sheets.

Panel (c) Cumulative curves of land loss since sea level rose 26 ky ago (bars; left axis) and rates of areas lost (km^2) per 500 years (dots; right axis). Land loss and rates of land loss were not synchronous with shelf expansion. While in Sunda most land loss occurred between 15 and 10 ky, both in the Banda Arc shelf region and the Caribbean the period of land loss was more extensive spanning from 17 to 7 ky and 15 to 6 ky BP. During these periods of sea transgression, coastal land drowned, some islands drowned entirely, and all islands contracted. The most extreme land losses occurred in the Sunda region with more than 2.6×10^6 km^2 lost, an area equivalent to nearly a third of Australia; the Caribbean lost 0.55×10^6 km^2, an area equivalent to the size of Madagascar, and the Banda Arc shelfs region lost 2.6×10^5 km^2, an area grossly equal to the United Kingdom.

Panel (d) Coastal retreat rates, maximum percentile (97,5% - 100%) coastal retreat rates in m / yr within the three study areas. The average coastal retreat rates in the Sunda region from 26 to 6 ky were about 11 m / y, locally exceeding 100 m / y, with three peaks at 15 to 14.5 ky, at 13 ky and 11.5 ky, two peaks of which roughly coincide with global sea level rise pulses at 15 ky during the Bölling-Allerød interstadial warming and 11 ky at the onset of the Holocene warming (Fig. 1a). Around the Caribbean coastal lowlands, the average coastal retreat rates were nearly 7 m per year between the LGM and 500 years ago. The highest coastal retreat rates at the Caribbean exceeded 15 m / y, which persisted longer than in Sunda, from 15 ky up to as recent as 500 years ago. Remarkably, in the Caribbean, geophysically mediated relative sea level rise effects culminated to its highest recorded coastal retreat rates of over 30 m / y as recently as between 3 ky to 500 y ago, which is related to the shallow bathymetry and to local subsidence. In Banda Arc region coastal retreat rates were on average 6.5 m / y, and maxima of 10 m / y occurred between 19.5 ky until as recent as 500 years ago.
